# Systematic analysis of the prognostic value and immunological function of LTBR in human cancer

**DOI:** 10.18632/aging.205356

**Published:** 2024-01-03

**Authors:** Yinteng Wu, Shijian Zhao, Wenliang Guo, Ying Liu, Marìa Del Mar Requena Mullor, Raquel Alarcòn Rodrìguez, Ruqiong Wei

**Affiliations:** 1 Department of Orthopedic and Trauma Surgery, The First Affiliated Hospital of Guangxi Medical University, Nanning, Guangxi 530021, China; 2 Department of Cardiovascular Medicine, The First Affiliated Hospital of Guangxi Medical University, Nanning, Guangxi 530021, China; 3 Department of Rehabilitation Medicine, The Eighth Affiliated Hospital of Guangxi Medical University, Guigang, Guangxi 537100, China; 4 Department of Rehabilitation Medicine, The First Affiliated Hospital of Guangxi Medical University, Nanning, Guangxi 530021, China; 5 Faculty of Health Sciences, University of Almerìa, La Cañada 04120, Almeria, Spain

**Keywords:** lymphotoxin beta receptor (LTBR), pan-cancer, immune microenvironment, T cell, drug sensitivity

## Abstract

Lymphotoxin beta receptor (LTBR) is a positive T cell proliferation regulator gene. It is closely associated with the tumor immune microenvironment. However, its role in cancer and immunotherapy is unclear. Firstly, the expression level and prognostic value of LTBR were analyzed. Secondly, the expression of LTBR in clinical stages, immune subtypes, and molecular subtypes was analyzed. The correlation between LTBR and immune regulatory genes, immune checkpoint genes, and RNA modification genes was then analyzed. Correlations between LTBR and immune cells, scores, cancer-related functional status, tumor stemness index, mismatch repair (MMR) genes, and DNA methyltransferase were also analyzed. In addition, we analyzed the role of LTBR in DNA methylation, mutational status, tumor mutation burden (TMB), and microsatellite instability (MSI). Gene Ontology (GO), Kyoto Encyclopedia of Genes and Genomes (KEGG), and Gene Set Enrichment Analysis (GSEA) were used to explore the role of LTBR in pan-cancer. Finally, the drugs associated with LTBR were analyzed. The expression of LTBR was confirmed using quantitative real-time PCR and Western blot. LTBR is significantly overexpressed in most cancers and is associated with low patient survival. In addition, LTBR expression was strongly correlated with immune cells, score, cancer-related functional status, tumor stemness index, MMR genes, DNA methyltransferase, DNA methylation, mutational status, TMB, and MSI. Enrichment analysis revealed that LTBR was associated with apoptosis, necroptosis, and immune-related pathways. Finally, multiple drugs targeting LTBR were identified. LTBR is overexpressed in several tumors and is associated with a poor prognosis. It is related to immune-related genes and immune cell infiltration.

## INTRODUCTION

Each aspect of the immune system is deeply involved in cancer development and progression [1]. Molecularly targeted therapies are advancing rapidly, and transcriptional analysis provides significant opportunities to understand the complexity of tumors, including the tumor microenvironment (TME), which plays a crucial role in cancer progression and treatment [2, 3]. Tumor-infiltrating immune cells have the ability to exert both pro- and antitumor effects, significantly impacting tumor progression and the efficacy of anti-cancer therapy [4, 5]. Immunotherapy has emerged as a promising approach for advanced cancers, offering breakthrough potential in tumor treatment [6]. However, the effectiveness of immune checkpoint inhibitors (ICIs), which have revolutionized cancer therapy, is limited to a select group of patients [7]. Many studies confirm that biomarkers are associated with disease progression [8–11]. In particular, immune-related genes are often closely associated with tumor progression [12, 13]. Thus, deciphering the tumor immune microenvironment signature may elucidate the potential mechanisms by which targeted therapies and immunotherapies generate therapeutic resistance. They have the potential to enhance the customization of targeted and immunotherapeutic approaches.

Lymphotoxin (LT), a tumor necrosis factor superfamily member, plays an important role in lymphoid organs [14]. Lymphocytes mainly produce it and induce LT is primarily produced by lymphocytes and causes the development of secondary lymphoid tissues, including lymph nodes and intestinal lymphoid follicles [15]. It has two subunits (LTα and LTβ) [16]. The LTβ receptor (LTBR) may be a critical factor in lymph node formation [17]. LT and/or LIGH ligands involve the LTBR signaling pathway and play a crucial role in developing and functioning high ECs (HECs) of LT and/or LIGH [18]. LTBR is expressed on various cell types including follicular dendritic cells (FDCs), DCs, macrophages, and stromal cells. Its expression leads to the upregulation of proinflammatory mediators, adhesion molecules, and chemokines such as CCL19, CCL21, and CXCL13 [19]. The ligand lymphotoxin α1β2 (LTα1β2) and its receptor, LTBR, have a crucial role in establishing and regulating the immune system by facilitating close communication between lymphocytes and stromal cells [20]. The therapeutic potential of LTBR-Ig has been demonstrated in multiple mouse models of autoimmune diseases including rheumatoid arthritis, colitis, experimental autoimmune encephalomyelitis (EAE), and advanced type 1 diabetes [21].

Overexpression of LTBR in T cells results in significant transcriptional and epigenomic remodeling. This leads to enhanced T cell effector function and resilience against failure in a chronic stimulatory environment. These effects are mediated through the structural activation of the canonical NF-κB pathway [22]. In a recent study, after cloning LTBR and expressing it in two T cell subsets, CD4+ and CD8+, researchers observed that it had the most pronounced effect on T cell function, increasing the secretion of multiple cytokines in CD4+ and CD8+ T cells by more than 5-fold. A genome-scale screen for synthetic LTBR signaling is involved in the host response to infection, regulates the acute inflammatory response, and mediates tumor cell apoptosis. Thus, LTBR signaling is involved in innate and acquired immune responses [23]. In addition, LTBR signaling has been shown to affect the development of various tumors, and inhibition of its expression can have an anti-tumor effect [24]. LTBR affects the survival time of patients with colorectal tumors by inducing the expression of IL-22 binding protein (IL-22BP) [25]. Lymphotoxins produced by cancer cells activate the LTBR-NF-κB signaling pathway in stromal fibroblasts, leading to the expression of chemokines. The therapeutic potential of targeting the lymphotoxin-LTBR and CXCL11-CXCR3 signaling pathways has been demonstrated in ovarian cancer [26]. Due to its association with immunity, LTBR and its ligands have gained attention as promising targets for the treatment of immune diseases and cancers.

In this study, we conducted a comprehensive analysis of LTBR expression, its prognostic implications, and its associations with clinical staging, immune subtypes, and molecular subtypes. We also investigated the relationship between LTBR and immunomodulatory genes, immune checkpoint genes, RNA modification genes, immune cell infiltration score, immune cells, DNA mismatch repair (MMR) genes, and DNA methylation transferases. Additionally, we explored the correlation between LTBR and cancer-related functional status at the single-cell level. The impact of LTBR on DNA methylation, mutational status, tumor mutational burden (TMB), and microsatellite instability (MSI) was examined. To uncover cancer-associated pathways associated with LTBR expression, we utilized Gene Ontology (GO), Kyoto Encyclopedia of Genes and Genomes (KEGG), and gene set enrichment analysis (GSEA). In addition, we also analyzed LTBR at the pan-cancer level to drug sensitivity.

## RESULTS

### Analysis of LTBR expression levels

**Figure 1:**
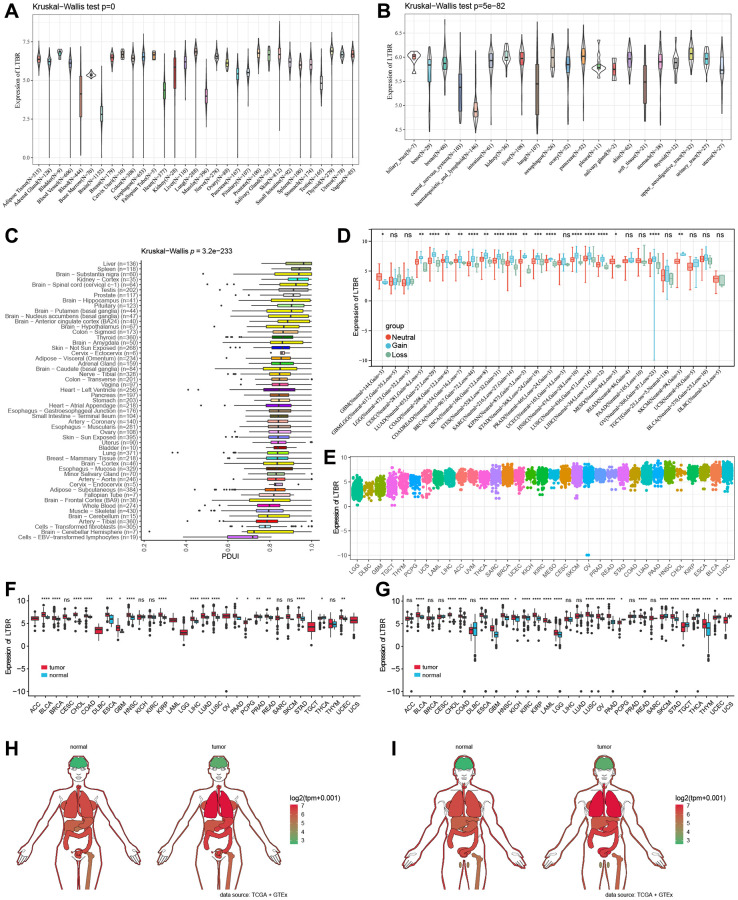
**LTBR mRNA expression.** (**A**) LTBR expression in 31 normal tissues and (**B**) 21 tumor cell lines. (**C**) APA landscape of LTBR in individual normal tissues. (**D**) Differential CNV expression levels of LTBR in individual tumors. (**E**) LTBR mRNA expression in tumor tissues from TCGA database. (**F**) Expression levels of LTBR in TCGA. (**G**) Combined GTEx database and TCGA analysis of LTBR expression levels. Anatomical maps of LTBR gene expression profiles in all tumor samples and normal tissues in females (**H**) and males (**I**).

**Figure 2:**
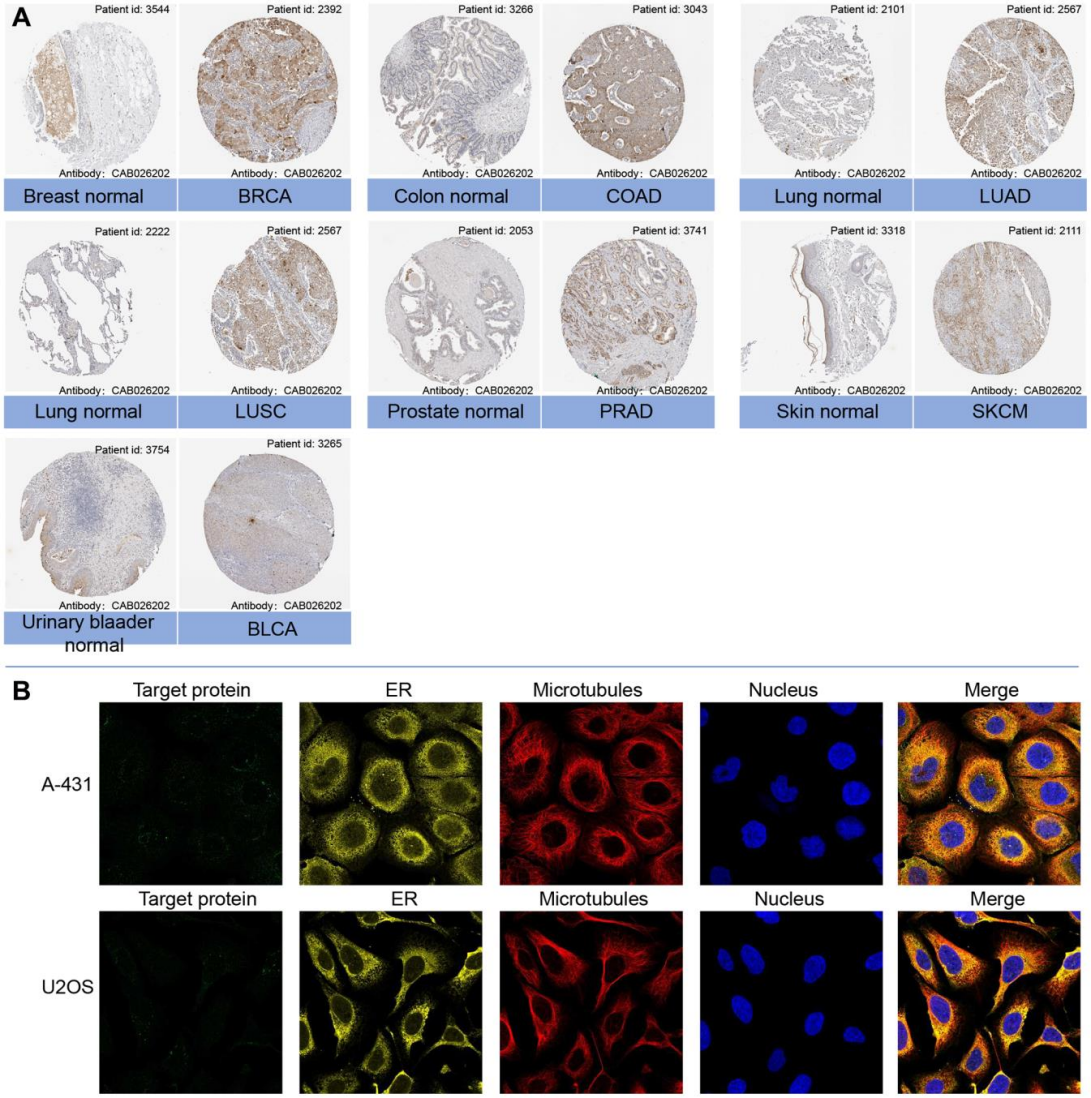
(**A**) Representative immunohistochemical staining (IHC) of LTBR in various normal (left) and tumor (right) tissues from Human Protein Atlas. (**B**) Protein subcellular localization the immunofluorescence images of LTBR protein from Human Protein Atlas.

The mRNA expression levels of LTBR were examined in various organs using data from the GTEx database, which encompassed diverse tissues from healthy individuals (Figure 1A). In making comparisons between tumor cell lines, we found that LTBR had high expression levels in the upper aerodigestive tract, pancreas and kidney tumor cell lines, as well as low expression levels in the central nervous system, haematopoietic and lymphoid tumor cell lines (Figure 1B). The findings indicated that LTBR expression was relatively consistent across different tumor cell lines. Figure 1C depicts the alternative polyadenylation (APA) profile of LTBR in each normal tissue. We looked at how much LTBR was expressed in each tumor. By analyzing copy number data and gene expression data in the samples, we observed significant differences in 18 tumors (Figure 1D). The results showed that LTBR had the highest LUSC, BLCA, and ESCA expression levels and the lowest in LGG, DLBC, and GBM (Figure 1E). Subsequently, the expression levels of LTBR in tumor and normal tissues were assessed utilizing data from the TCGA database. The analysis unveiled a significant upregulation of LTBR expression in 16 different cancer types (Figure 1F). Next, we integrated the GTEx database to compare LTBR expression between tumor tissues and normal tissues across 27 different types of cancer. The analysis demonstrated that LTBR exhibited increased expression levels in 17 cancer types, suggesting its potential role as an oncogene in these specific cancers (Figure 1G). Subsequently, we examined the expression patterns of LTBR in active body maps using the GEPIA dataset, which revealed distinct expression differences between tumor tissues and their corresponding normal tissues (Figure 1H, 1I). To further validate these findings, IHC results of BRCA, COAD, LUAD, LUSC, PRAD, SKCM, BLCA, and normal tissues were examined (Figure 2A), which consistently showed high expression of LTBR in various tumors. The cancer cells’ immunofluorescence (IF) images indicated that LTBR is mainly located in the Golgi apparatus (Figure 2B). Figure 2A, 2B available from https://www.proteinatlas.org/search/LTBR.

### Prognostic value of LTBR

**Figure 3:**
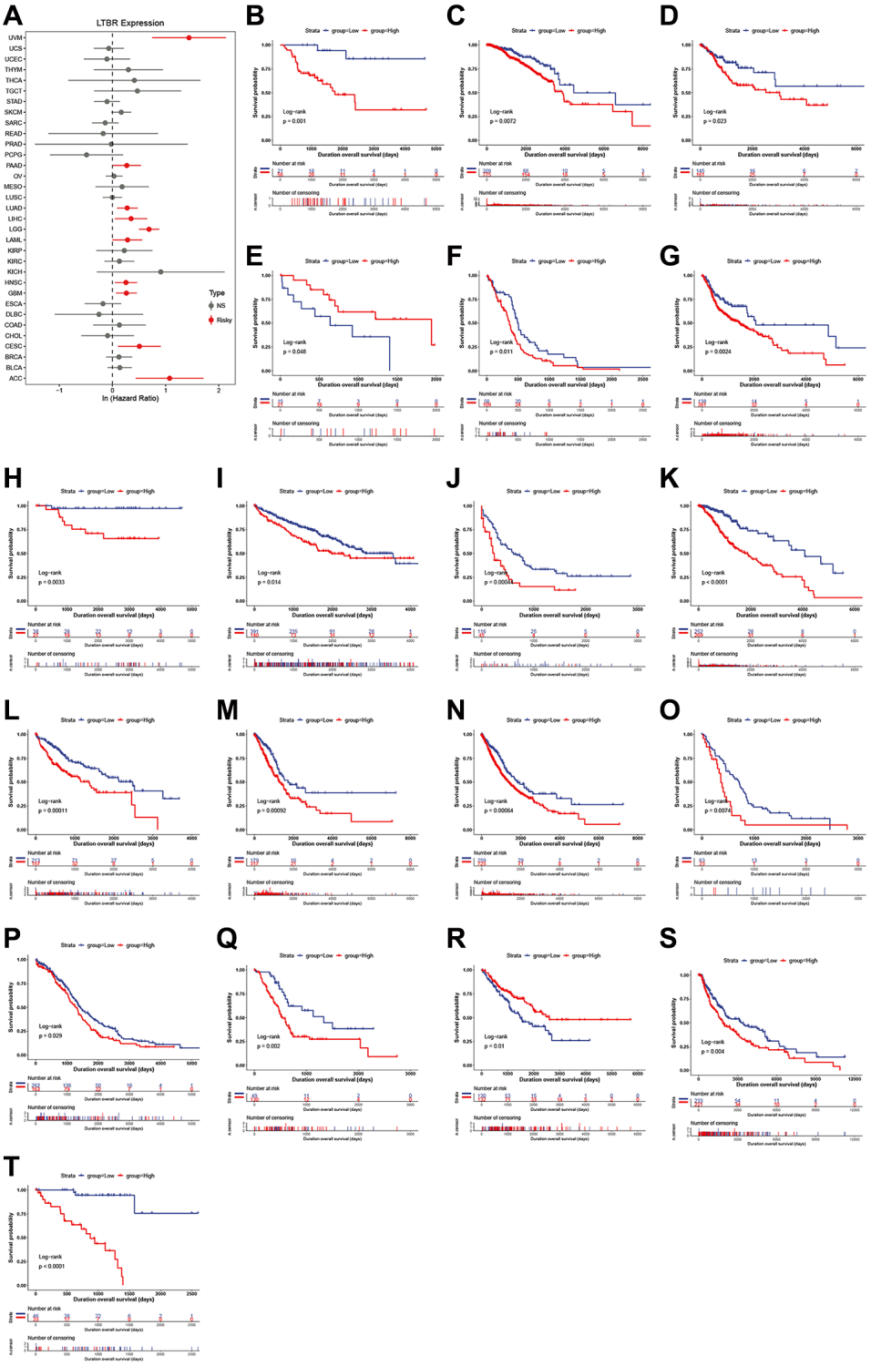
**The relationship of LTBR expression with patients’ OS.** (**A**) Forest plots of hazard ratios of LTBR in 33 cancer types. Log-rank test OS curves for patients stratified by different expression levels of LTBR in (**B**) ACC, (**C**) BRCA, (**D**) CESC, (**E**) CHOL, (**F**) GBM, (**G**) HNSC, (**H**) KICH, (**I**) KIRC, (**J**) LAML, (**K**) LGG, (**L**) LIHC, (**M**) LUAD, (**N**) LUNG, (**O**) MESO, (**P**) OV, (**Q**) PAAD, (**R**) SARC, (**S**) SKCM, (**T**) UVM.

**Figure 4:**
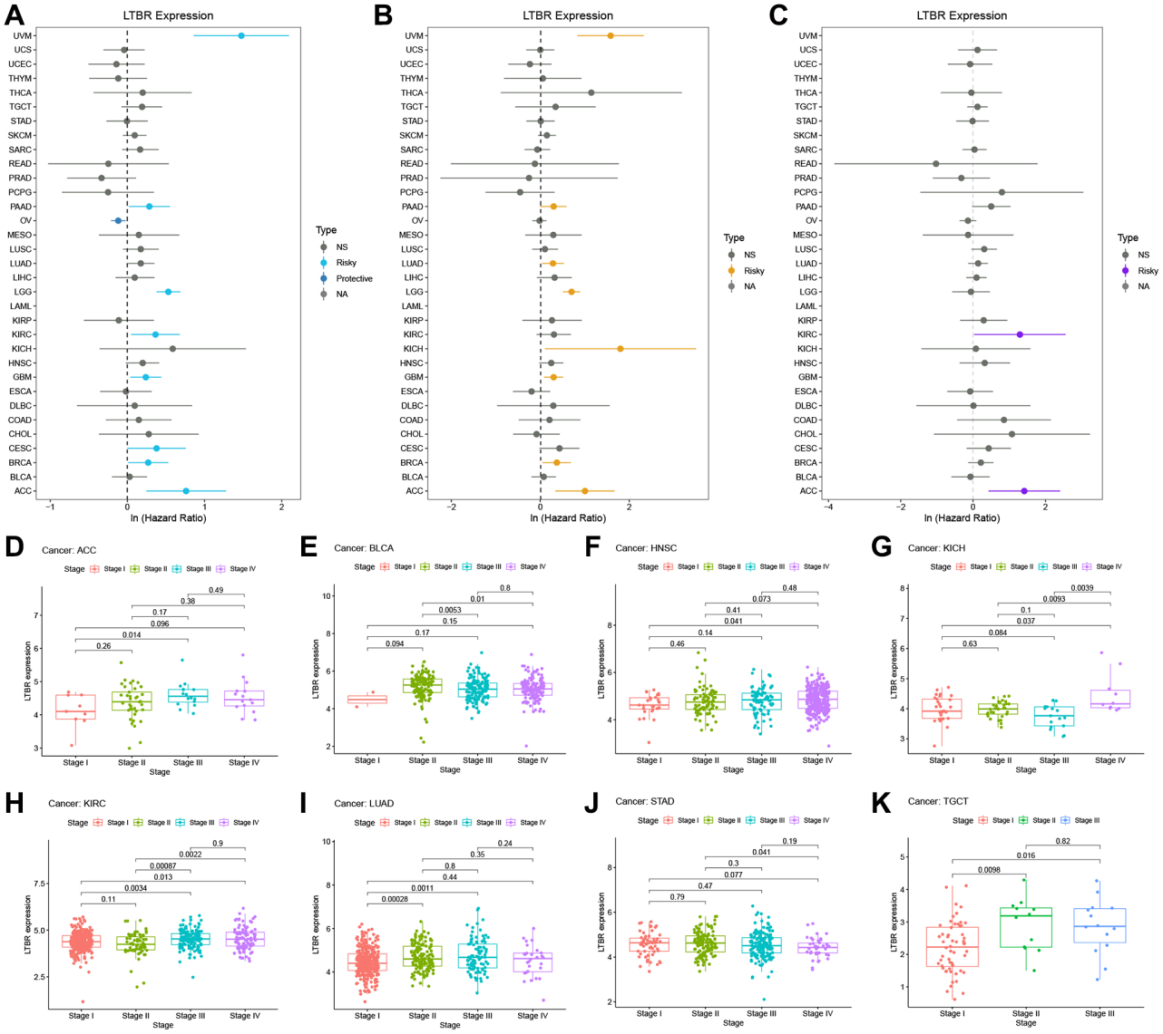
**The relationship of LTBR expression with patients’ PFI, DSS, DFI, and clinical stage.** Forest plots of hazard ratios of LTBR in (**A**) PFI, (**B**) DSS, and (**C**) DFI. (**D**–**K**) Pan-cancer differential expression of LTBR in clinical stages.

One-way Cox regression analysis and Log-rank test survival analysis were utilized to assess the prognostic value of LTBR in cancer patients. UniCox results indicated that LTBR has been recognized as a significant risk factor for overall survival (OS) in patients with UVM, PAAD, LUAD, LIHC, LGG, LAML, HNSC, GBM, CESC, and ACC (Figure 3A). Log-rank test OS analysis demonstrated that elevated P4HA1 expression predicted worsening OS in patients with ACC, CESC, GBM, HNSC, LAML, LGG, LIHC, LUAD, PAAD, and UVM (Figure 3B–3T). Furthermore, the predictive value of LTBR in DSI, DSS, and PFI using one-way Cox regression analysis. As shown in Figure 4A, LTBR was identified as a risk factor for PFI in ACC, BRCA, CESC, GBM, KIRC, LGG, PAAD, and UVM patients, while it exhibited a protective effect in OV patients. LTBR emerged as a risk factor for disease-specific survival (DSS) in ACC, BRCA, GBM, KICH, LGG, LUAD, PAAD, and UVM patients (Figure 4B). Additionally, LTBR was identified as a risk factor for disease-free interval (DFI) in ACC and KIRC patients (Figure 4C).

### Value of LTBR in clinical staging, immune subtypes, and molecular subtypes

**Figure 5:**
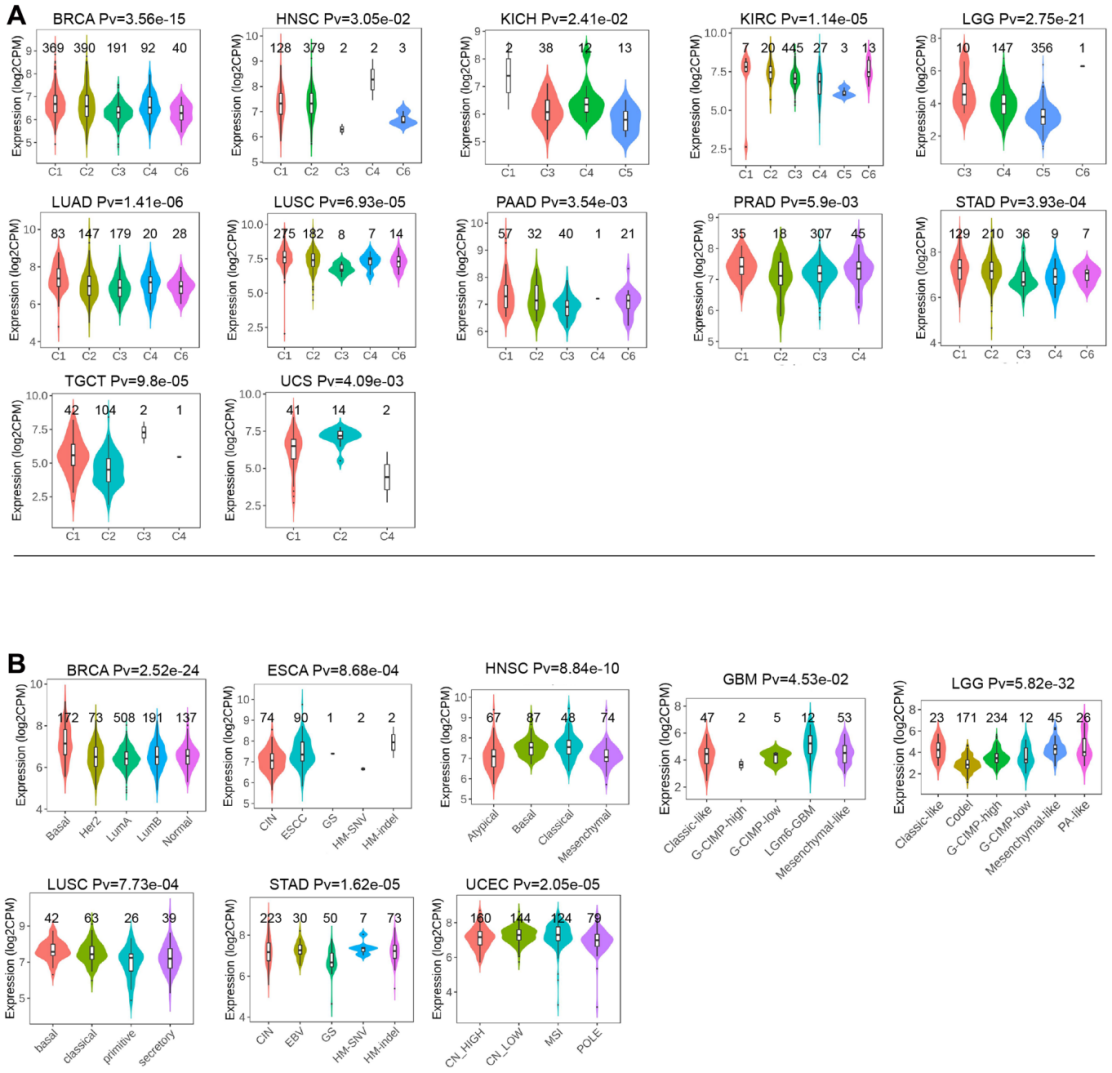
(**A**) The relationship of LTBR expression with immune subtypes in BRCA, HNSC, KICH, KIRC, KIRP, LGG, LUAD, LUSC, PAAD, PRAD, STAD, TGCT, and UCS. (**B**) The differences of LTBR expression levels among distinctive methyltransferase in BRCA, ESCA, HNSC, GBM, LGG, LUSC, STAD, and UCEC.

Moreover, we examined the expression of LTBR across various stages of different cancers according to WHO classification. Our analysis revealed that LTBR exhibited elevated expression in advanced stages of ACC, HNSC, KICH, KIRC, LUAD, and TGCT, while demonstrating lower expression in higher stages of BLCA and STAD (Figure 4D–4K). LTBR showed significant associations with immune subtypes in 12 tumors (Figure 5A). Regarding molecular subtype, LTBR was substantial in 8 tumors (Figure 5B).

### Correlation analysis of LTBR with immunomodulatory genes, immune checkpoint genes, and RNA modifier genes

**Figure 6:**
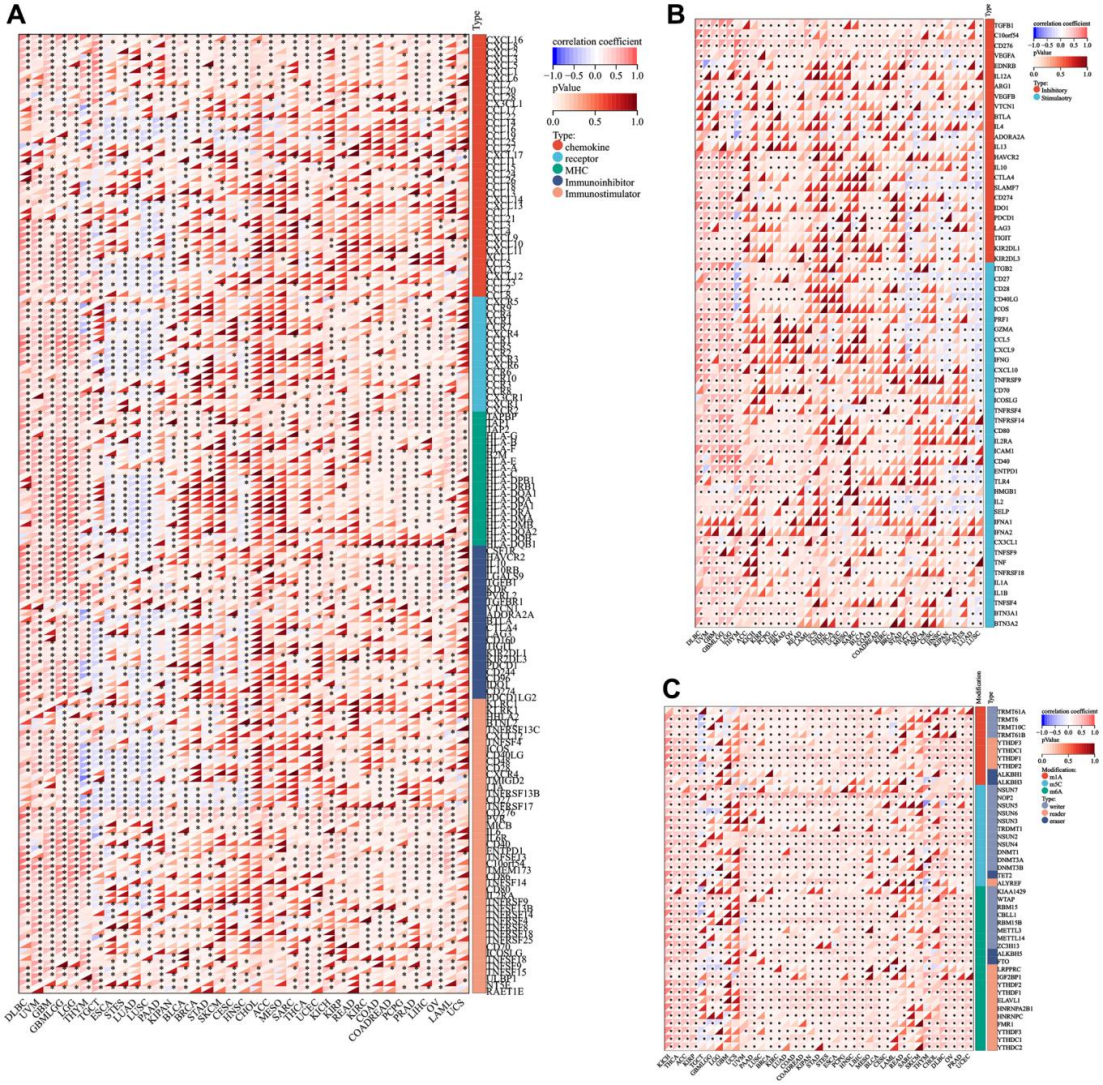
**Co-expression of LTBR with immune-associated genes.** (**A**) Co-expression between LTBR and immunoregulatory genes. (**B**) Immune checkpoint genes. (**C**) RNA modifier genes. ^*^p < 0.05, ^**^p < 0.01, and ^***^p < 0.001.

LTBR was positively correlated with immunomodulatory genes in most tumors. LTBR exhibited a positive correlation with CXCL16, CXCL8, CXCL3, CXCL2, CXCL6, CCL20, TAPBP, TAP1, TAP2, HLA-G, HLA-B, IL10RB, LGALS9, TGFB1, KDR, PVRL2 TGFBR1, CD276, PVR, MICB, IL6, IL6R, C10orf54, TMEM173, TNFRSF14, TNFSF9, TNFSF15, ULBP1, NT5E, and RAET1E had significant positive correlations in most tumors (Figure 6A). In terms of immune checkpoint genes, LTBR had significant positive correlations with TGFB1, C10orf54, CD276, VEGFA, EDNRB, CX3CL1, TNFSF9, TNF, TNFRSF18, IL1A, IL1B, TNFSF4, BTN3A1, BTN3A2, and HMGB1 in most tumors (Figure 6B). In the majority of tumors, LTBR displayed significant correlations with m1A, m5c, and m6A-related genes (Figure 6C).

### Immuno-infiltration analysis

**Figure 7:**
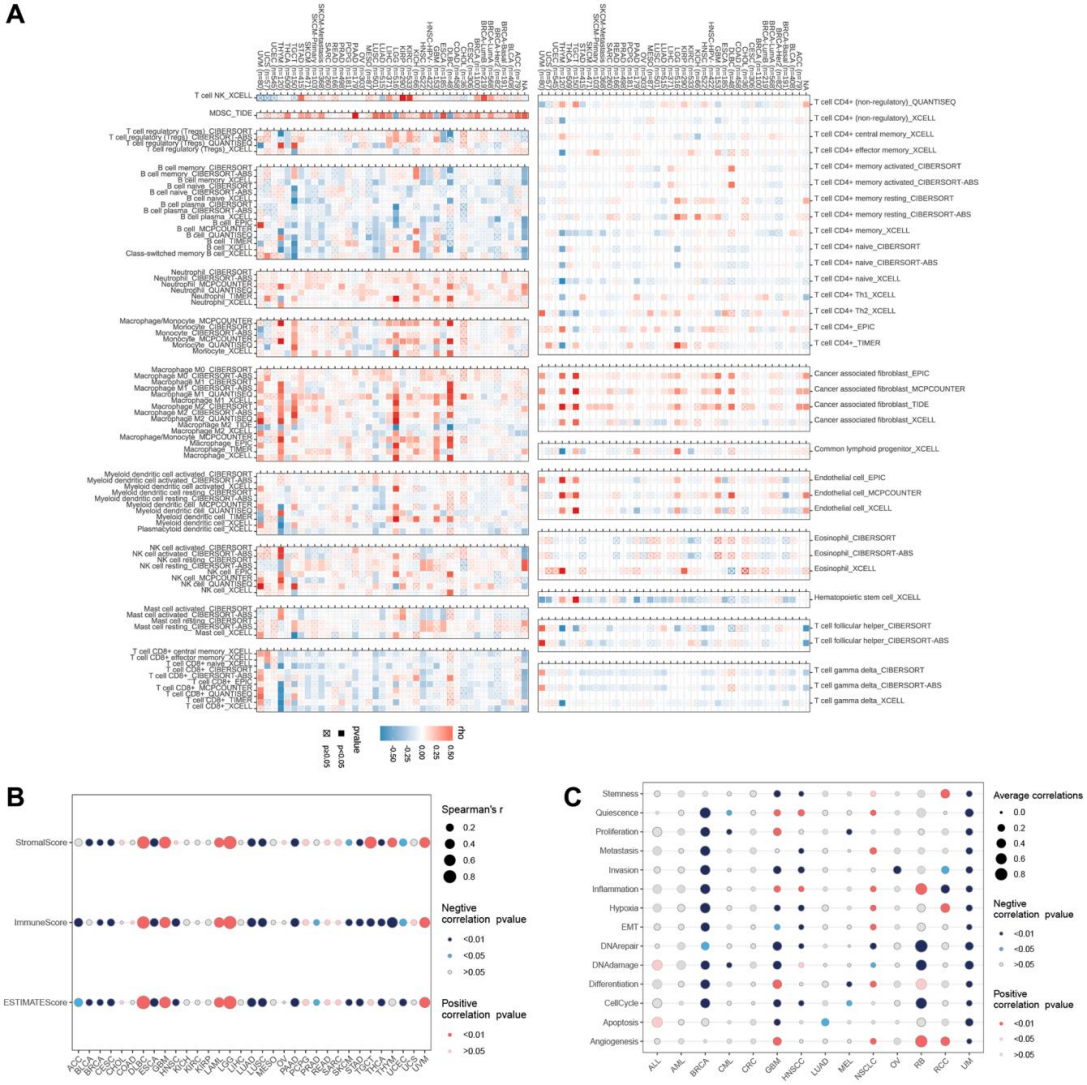
(**A**) The relationship of LTBR expression with immune cell infiltration analysis. The relationship of LTBR expression with (**B**) immune infiltration. (**C**) Cancer-related functional status analysis.

Additionally, the correlation heat map demonstrated the close associations between LTBR and various immune cells at the pan-cancer level (Figure 7A). For ESTIMATEScore, ImmuneScore, and StromalScore (Figure 7B), LTBR had substantial positive correlations with all three scores in DLBC, GBM, LAML, LG, and UVM; LTBR had significant positive correlations with BRCA, CESC, LUAD, LUSC, PAAD, SKCM, STAD THCA, and UCEC were significantly negatively correlated with all three scores. Regarding cancer-related functional status (Figure 7C), LTBR and UM were both significantly negatively correlated; LTBR exhibited significant negative correlations with 11 cancer-related functional states in BRCA and significant positive correlations with eight cancer-related functional statuses in NSCLC.

### Correlation analysis of LTBR and tumor stemness index

**Figure 8:**
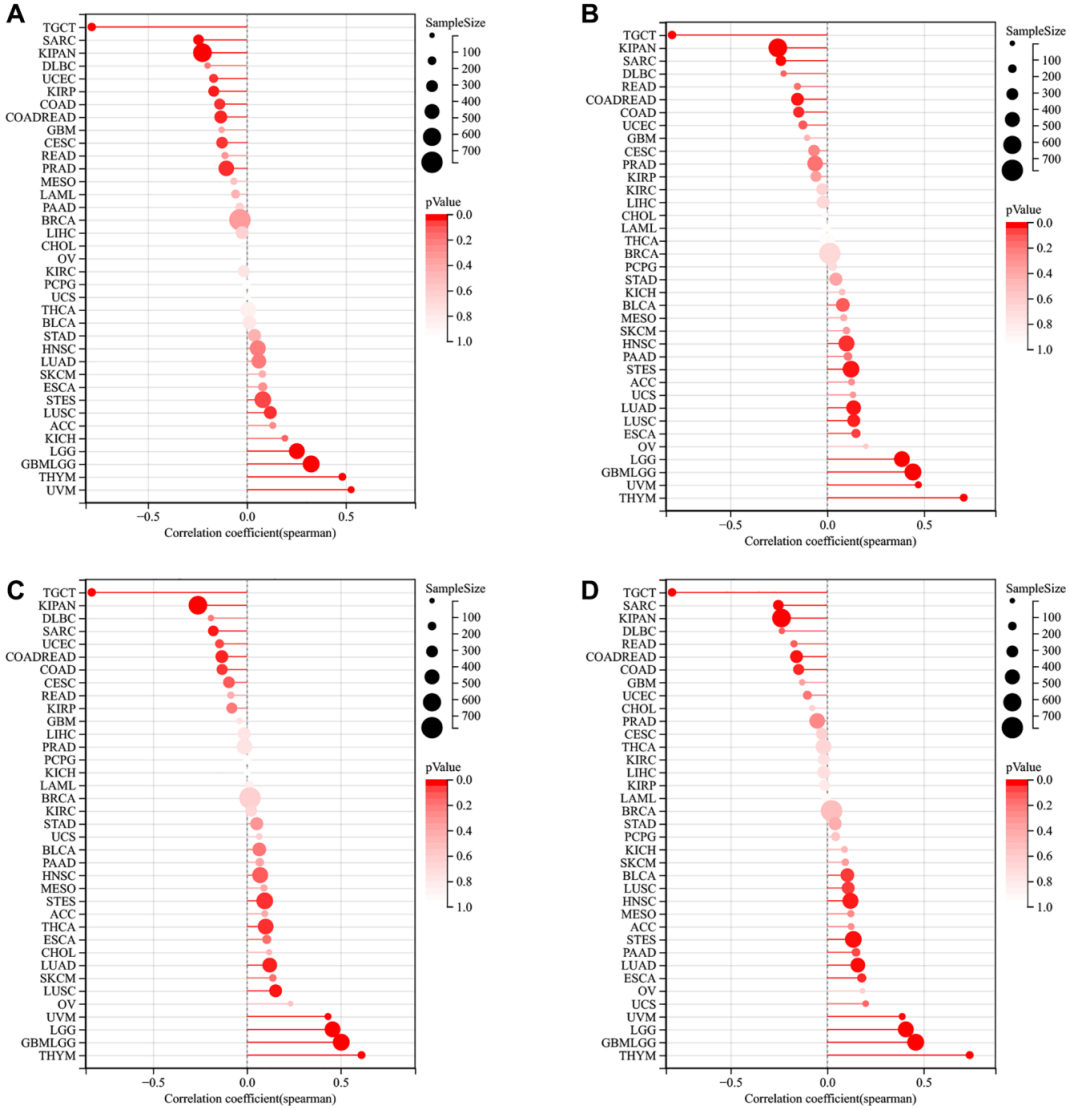
The relationship of LTBR expression with tumor stemness index from (**A**) DMPss, (**B**) DNAss, (**C**) ENHss, (**D**) EREG-METHss algorithm.

According to the results of DMPss (Figure 8A), DNAss (Figure 8B), ENHss (Figure 8C), and EREG-METHss (Figure 8D), the correlation between LTBR and stemness index was consistent across multiple tumors, such as LTBR was significantly positively correlated with TGCT, SARC, KIPAN, DLBC, UCEC, KIRP, and COAD, significantly negatively correlated with STES, LUSC, ACC, KICH, LGG, GBMLGG, THYM, and UVM.

### TMB, MSI, mutation, and methylation analysis

**Figure 9:**
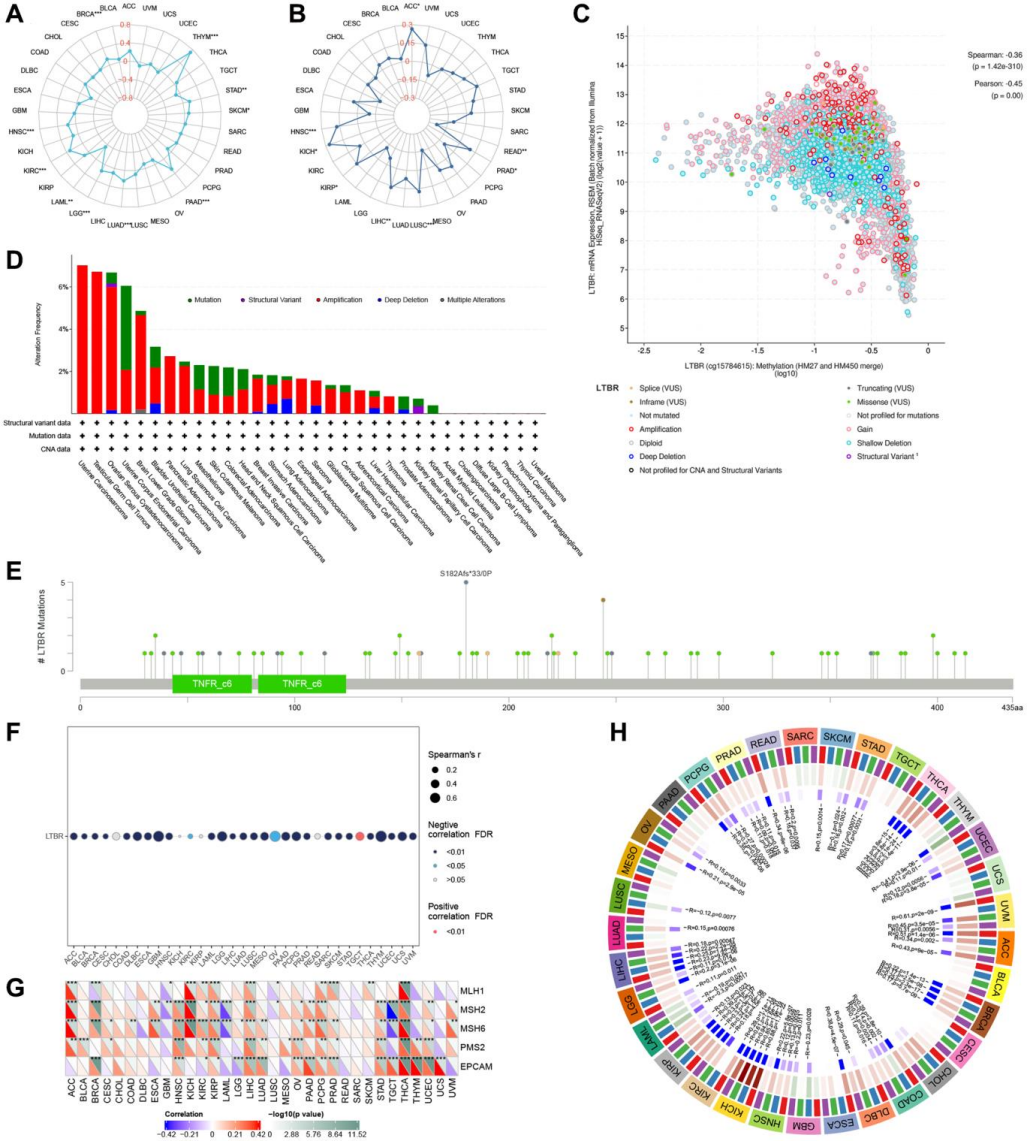
The relationship of LTBR expression with (**A**) TMB and (**B**) MSI. (**C**) LTBR mRNA expression vs. methylation. (**D**) The relative frequency of each mutation type and (**E**) mutation site. (**F**) The relationship of LTBR expression with methylation. (**G**) MMR genes in human pan-cancer (^*^P < 0.05, ^**^P < 0.01, ^***^P < 0.001). Correlation analysis of LTBR expression with (**H**) DNA methyltransferases (DNMT1: red; DNMT2: blue; DNMT3A: green; DNMT3B: purple).

Regarding TMB (Figure 9A), LTBR and THYM, STAD, SKCM, PAAD, LUAD, LGG, KIRC, HNSC, and BRCA were significantly positively; LTBR and LAML were significantly negatively correlated. Regarding MSI (Figure 9B), LTBR and ACC, PRAD, LUSC, LIHC, KIRP, KICH, and HNSC were significantly positively correlated; LTBR and READ were significantly negatively correlated. At the pan-cancer level, LTBR significantly correlated with methylation (Figure 9C). Amplification was predominant among all mutation types, and the highest LTBR mutation frequency was observed in Uterine Carcinosarcoma (Figure 9D). The mutation site information of LTBR is shown in Figure 9E. Among them, LTBR demonstrated significant negative correlations with DNA methylation in 28 cases (Figure 9F).

### MMR genes and DNA methyltransferase analysis

We also looked at the relationship between LTBR expression and mutation levels in MMR genes. The findings revealed significant correlations between LTBR expression and the mutation levels of five MMR genes (MLH1, MSH2, MSH6, PMS2, EPCAM) in pan-cancer tissues (Figure 9G). Additionally, to investigate the role of LTBR in tumorigenesis, we analyzed the correlation between LTBR expression and four DNA methyltransferases. The results demonstrated significant associations between LTBR expression and at least one DNA methyltransferase, except for CESC, CHOL, MESO, and PAAD (Figure 9H).

### GO, KEGG, and GSEA results

**Figure 10:**
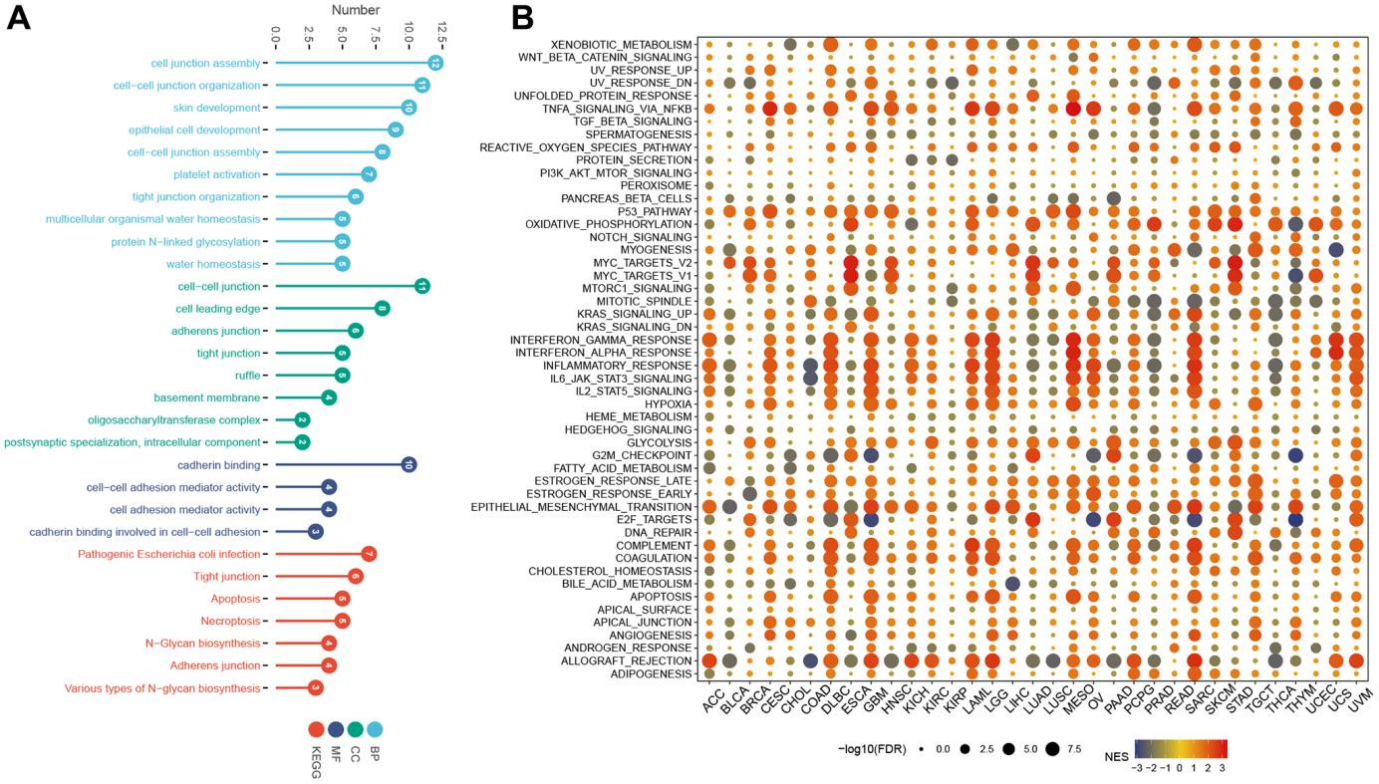
**GO, KEGG, and GSEA analysis.** (**A**) GO, and KEGG analysis. (**B**) GSEA of LTBR in the hallmarks gene set.

The GO analysis revealed significant enrichment of genes in processes related to cell junction assembly, cell-cell junction organization, skin development, cell-cell junction, cell leading edge, cadherin binding, and cell-cell adhesion mediator activity (Figure 10A); The KEGG analysis revealed a significant increase in genes associated with Pathogenic Escherichia coli infection, Tight junction, apoptosis, necroptosis, N-Glycan biosynthesis, Adherens junction and Various types of N-glycan biosynthesis. GSEA results showed that in most tumors, tnfa_signaling_via_nfkb, p53_pathway, oxidative_phosphorylation, interferon_gamma_response, interferon_alpha_response, inflammatory_response, epithelial_mesenchymal_transition, and coagulation were activated (Figure 10B).

### Drug sensitivity analysis

**Figure 11:**
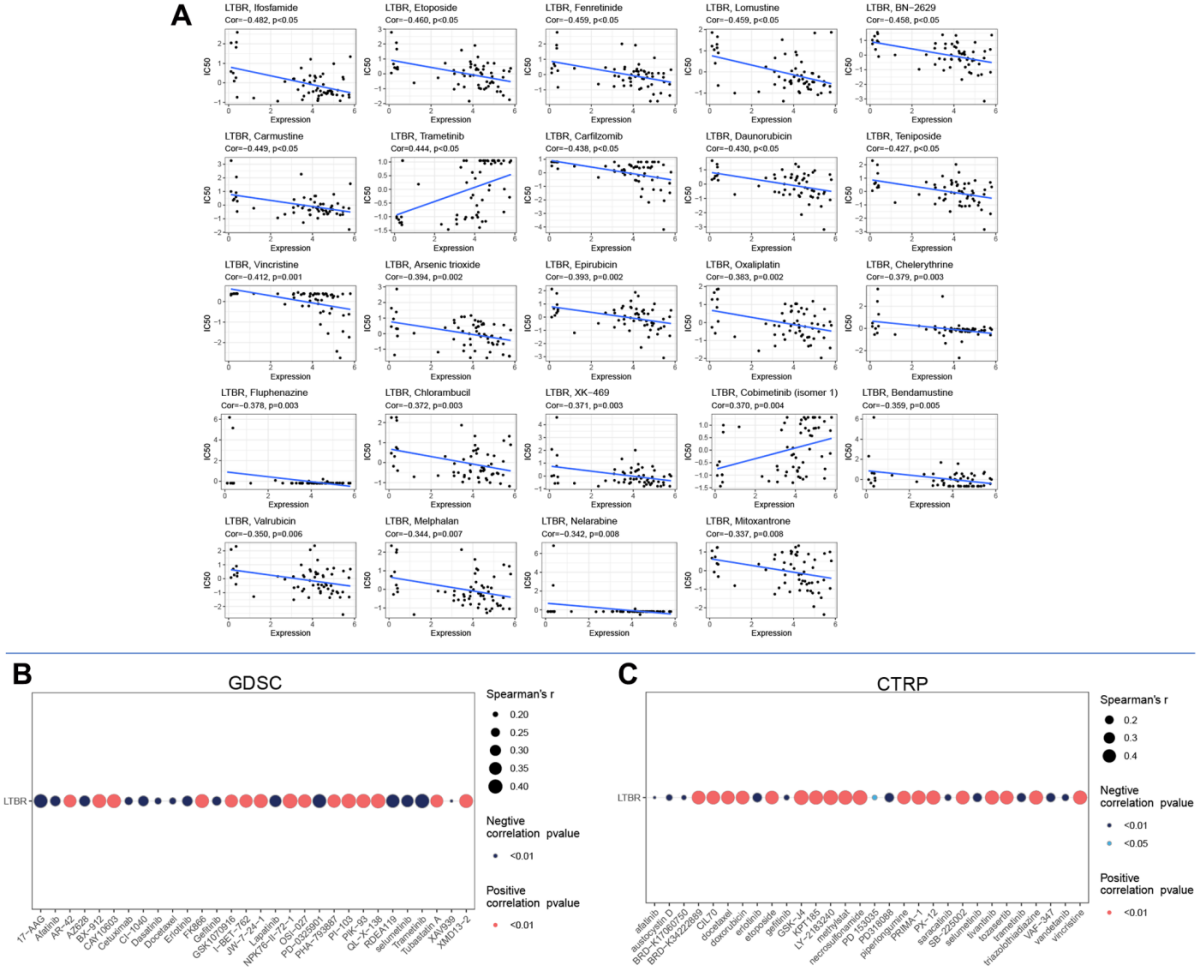
Drug sensitivity analysis in (**A**) cellMiner database, (**B**) GDSC database, and (**C**) CTRP database.

The expression of LTBR displayed a significant negative correlation with 22 drugs and a significant positive correlation with two drugs (Figure 11A). In addition, based on the GDSC (Figure 11B) and CTRP (Figure 11C) results, we identified multiple drugs significantly positively correlated with LTBR, which expands the scope of developing potential drugs targeting LTBR.

### Experimental verification

**Figure 12:**
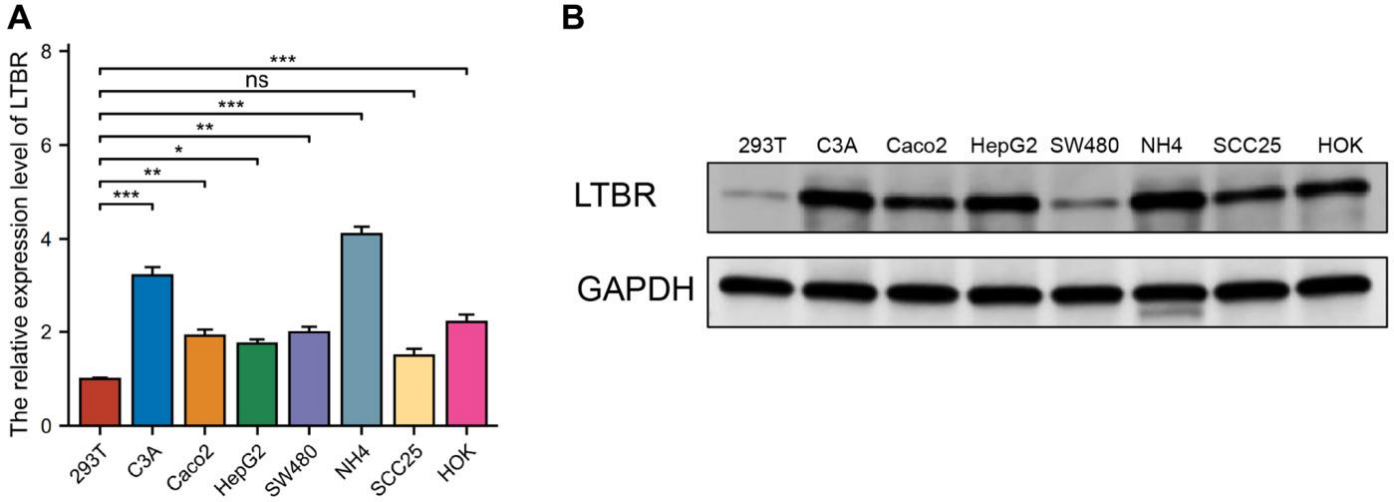
**Validation of expression of the LTBR gene.** (**A**) RT-qPCR results revealed elevated levels of LTBR expression in most cancer cell lines. (**B**) Western blot results indicated an increased protein level of LTBR across the majority of pan-cancer cell lines, compared to the normal cell lines.

A selection of normal (293T, HOK) and cancerous (C3A, Caco2, HepG2, SW480, NH4, SCC25) cell lines were utilized to detect the expression of LTBR, identified as a key risk gene in previous screenings. We began by validating the expression of LTBR at the transcriptome level using real-time quantitative polymerase chain reaction (RT-qPCR). It was found that LTBR exhibited a higher degree of expression in cancer cell lines compared to normal ones, yet this expression was notably absent in certain hepatoma cells (Figure 12A). Subsequently, the expression of the associated proteins was confirmed in the aforementioned cell lines at the proteomic level via Western blotting (Figure 12B). The data indicated that these proteins were relatively overexpressed in more cancer cell lines.

## DISCUSSION

While immune checkpoint inhibition has revolutionized cancer therapy, sustained responses are observed in only a small fraction of patients, and these responses may come with the risk of severe toxicity [27]. As a result, many researchers are dedicated to exploring new therapeutic approaches for tumors [28, 29]. Using the patient’s T-cells, cell therapy has begun to revolutionize the treatment of several tumors [30]. Nevertheless, these cell therapy approaches’ response efficiency and cure rates require further improvement. However, most research on T-cell function has focused on negative regulators with functional deficiencies [31]. The researchers screened genome-wide libraries to identify various positive regulators of T cell function, particularly LTBR, that promote CD4+ and CD8+ T cell proliferation and activate the secretion of critical cytokines [22]. In this work, we looked into the expression of LTBR at multiple levels. The combined GTEx and TCGA databases indicated significant upregulation of LTBR expression in 17 tumors and especially low expressed in 6 tumors. In addition, Kaplan-Meier and univariate Cox regression analyses demonstrated that increased expression of LTBR was associated with unfavorable prognosis. It has been established that LTBR signaling pathway has an impact on hepatitis B virus (HBV) infection, hepatitis, and hepatocarcinogenesis [32]. LTBR in microenvironmental cells induces T-cell acute lymphoblastic leukemia with a cortical/mature immune phenotype [33]. Langerhans cells activate lymphatic endothelial cells through the LIGHT-LTBR signaling axis, thereby promoting dendritic cell migration or tumor cell metastasis [34]. Interference with the VEGF receptor-3 and the LTBR signaling pathways in high-grade b-cell lymphoma inhibited lymphoma angiogenesis [35]. The activation of LTB/LTBR stimulates the NIK-NF-κB2/RELB pathway, leading to enhanced migration of HNSCC cells mediated by met. This pathway holds potential as a therapeutic target [36]. Our findings are consistent with the oncogenic effect of abnormal LTBR expression observed in these investigations.

This study examined the potential association between LTBR expression and mutations, methylation, CNV, RNA modifier genes, MMR genes, and methyltransferases in all TCGA tumors. Genetic alterations also affected the mRNA expression of genomic genes [37]. The low incidence of LTBR mutations in tumors shows that LTBR gene alterations may not significantly contribute to cancer development. Another study found a correlation between CNV and gene mRNA expression and lower survival time [38]. LTBR was found to be significantly positively connected with CNV in the majority of tumors, indicating that LTBR and cancer patient prognosis are inextricably intertwined. Moreover, alterations in DNA methylation promote cancer progression [39]. LTBR was negatively correlated with methylation sites in most tumors, which explains the high expression of LTBR in most tumors. A previous study found that LTBR was negatively correlated with DNA methylation status [40]. Which further confirms our findings. Furthermore, in BRCA, KICH, KIRP, LIHC, and THCA, LTBR expression displayed a significant positive association with methyltransferases (DNMT1, DNMT2, DNMT3A, and DNMT3B). MMR genes play a crucial role in identifying and repairing various types of mutations during DNA replication, such as base substitutions, insertions, deletions, or mismatches [41]. Mutations or abnormalities in MMR genes cause the accumulation of genetic mistakes, which leads to genomic or microsatellite instability and eventual cancer [42]. Notably, LTBR expression has been linked to the expression of five MMR genes in human pan-cancer, particularly THCA, PGPC, and LIHC. mRNA modifications can regulate mRNA fate post-transcriptionally. Recent investigations have shown that n-methyladenosine (mA) is widely present in the internal sites of mRNAs, disrupting Watson-Crick base pairing and resulting in compromised gene expression [43]. For instance, abnormal m6A methylation can stimulate or repress the expression of target genes, influencing the development of breast, lung, liver, colorectal, leukemia, and glioblastoma [44]. Our study uncovered a positive correlation between LTBR and the expression of m1A, m5C, and m6A RNA modification regulators in the majority of tumors. These findings suggest that abnormal LTBR expression may contribute to tumorigenesis by regulating methylation, CNV, RNA modifier genes, MMR genes, and methyltransferases.

In recent years, an increasing body of research has established a close relationship between the immune status of tumors and the composition as well as invasiveness of cells within their microenvironment [45–47]. We found that LTBR was highly associated with ESTIMATEScore, ImmuneScore, and StromalScore in many cancers. TMB, a biomarker of immune checkpoint inhibitor response that reflects the total neoantigen load within the tumor, is strongly correlated with the efficacy of immunotherapy [48]. TMB has been used to assess gene mutations in cancer patients and thus to understand the effectiveness of immunotherapy in cancer [49]. MSI is also a critical biomarker that predicts the response to immune checkpoint inhibitors ICIs. The U.S. Food and Drug Administration (FDA) has approved high microsatellite instability (MSI-H) status or deletion mismatch repair (DMMR) as predictive biomarkers for guiding the therapeutic application of ICIs in certain types of cancer. Choosing Tumor Mutational Burden Wisely for Immunotherapy: correlation analysis revealed significant associations between LTBR and MSI or TMB in various cancers. Specifically, LTBR exhibited significant correlations with BRCA, THYM, STAD, SKCM, PAAD, LUAD, LGG, LAML, KIRC and HNSC for TMB, and with ACC, READ, PRAD, LUSC, and LIHC for MSI. LUSC, LIHC, KIRP, KICH, and HNSC were significantly correlated. LTBR demonstrated strong associations with immune regulatory genes and checkpoint genes in most tumors, suggesting its involvement in cancer progression and prognosis through interactions with the tumor microenvironment. Moreover, LTBR has been implicated in macrophage-driven inflammation in atherosclerotic lesions, potentially through enhanced ccl5-mediated monocyte recruitment [50]. Additionally, lymphotoxin derived from cancer cells can induce chemokine expression in stromal fibroblasts via the LTBR-NF-κB signaling pathway, while the lymphotoxin-LTBR and CXCL11-CXCR3 signaling pathways serve as therapeutic targets in ovarian cancer [26]. Furthermore, regulatory T cells (Tregs) express high levels of cell surface LTα1β2, which activates LTBR on lymphatic endothelial cells (LECs). This interaction regulates LEC adhesion molecules, intercellular junctions, and chemokines [51]. The LTBR on stromal cells is involved in the atypical NF-κB pathway. It mediates the expression of RelB-dependent homeostatic chemokines that direct naive lymphocyte homeostasis into secondary lymphoid organs (SLOs) [52]. Human LTBR, a member of the tumor necrosis factor receptor superfamily, plays critical roles in secondary lymphoid organ development, host defense, chemokine production, and apoptosis [53]. The crosstalk between TNFR-1 and LTBR results in elevated secretion of lymphogenic chemokine proteins. Additionally, the supernatants from SMCs activated by TNFR-1/LTBR enhance the migration of splenic T cells, B cells, and macrophages/dendritic cells [54]. The LTα1β2-LTBR signaling pathway plays a more important function in the maintenance of the thymic microenvironment, mainly through regulating tumor rejection antigen (TRA) and chemokine expression in medullary thymic epithelial cell (mTEC) (low) to effectively induce central tolerance [55]. These findings suggest a strong association between LTBR expression, immune infiltration, and patient prognosis.

T cells, as a crucial component of the tumor immune defense system, play a vital role in tumor immunity. T cells migrate through tissues searching for MHC-peptide complexes that activate their T cell receptors and sense numerous signals that warn them of malignancy. Thus, T cells are an essential component of immunotherapy [56]. Tumor-associated macrophages (TAMs), an important component of the tumor microenvironment (TME), function as central regulators of cancer-associated inflammation [57]. TAMs modulate tumor immunity by influencing the activity of other immune cells and secreting cytokines that interact with immune checkpoints [58, 59]. LTBR is required to migrate and select autoreactive T cells in the thymic medulla [60]. LTBR-dependent tertiary lymphoid tissue structures recruit and activate initial T cells in the islets [21]. Tregs exhibit high levels of cell surface LTα1β2, activating LTBR on lymphatic endothelial cells (LECs) and modulating the expression of LEC adhesion molecules, intercellular junctions, and chemokines [51]. TNFR-1/LTBR crosstalk increases the secretion of lymphatic-derived chemokine proteins. TNFR-1/LTBR-activated supernatants derived from SMCs strongly support the migration of splenic T cells, B cells, and macrophages/dendritic cells [54]. Overexpression of LTBR in T cells leads to extensive transcriptional and epigenomic remodeling, enhances T cell effector functions via structural activation of the canonical NF-κB pathway, and confers resistance to failure in a chronic stimulatory environment [22]. LTBR-ig-mediated suppression of the expansion and activity of PDPN LNSCs significantly reduces melanoma tumor growth and promotes the infiltration and proliferation of CD4 TILs [61]. The addition of LTBR also caused T cells to secrete more cytokines. The overexpression of LTBR in T cells induced the expression of numerous genes that enhance T cell function, thereby playing a crucial role in promoting the antitumor activity of T cells. Interestingly, LTBR is not normally expressed in T cells, highlighting the ability of genome-scale screens to find genes that activate a novel cellular program [22]. The LIGHT/LTBR axis is a major atherosclerotic major pathway, and its inactivation may reduce inflammation and macrophage proliferation associated with atherosclerotic burden in met/IR [62]. Differentiating CD169 macrophages in LN and spleen requires dual signaling from LTBR and RANK, related to immune response [63]. In the spleen, LTBR signaling is essential for various processes such as the development of B-cell follicles, follicular dendritic cells (FDCs), neutrophil recruitment, and maintenance of the marginal zone. Moreover, in adulthood, the LTBR signaling pathway plays a crucial role in maintaining the homeostasis of neutrophils, natural killer (NK) cells, and invariant natural killer T (iNKT) cells [64]. TNFR-1/LTBR crosstalk leads to increased secretion of lymphatic-derived chemokine proteins. TNFR-1/LTBR-activated SMC supernatants significantly support migrating splenic T cells, B cells, and macrophages/dendritic cells [54]. These studies emphasize the significance of LTBR’s connection with T cells and other immune cells, reiterating its position in the immunological milieu.

According to the results of our GSEA research, LTBR is intimately correlated with immune response-related activities such as TNF-a signaling via NF-κB, il2 stat5 signaling, inflammatory response, interferon alpha response, apoptosis in GO and KEGG analyses of genes similar to LTBR; we similarly found LTBR to be involved in apoptosis and necroptosis. In addition, for both GSEA and cancer-related functions at the single-cell level status, we found that LTBR exhibited associations with various processes in different cancers, including angiogenesis, apoptosis, DNA damage, DNA repair, cellular hypoxia, and inflammogenesis. Apoptosis, which is a programmed cell death process, plays a crucial role in eliminating cancer cells. It is mediated by multiple signaling pathways that are triggered by factors such as cellular stress, DNA damage, and immune surveillance [65]. However, apoptosis can suppress cancer and promote tumor growth [66]. By controlling RNA transcription and processing, DNA damage alters the cellular transcriptome. Such changes in gene expression in cancer cells can affect immune surveillance and cell death pathways [67]. Interestingly, researchers discovered that introducing cells undergoing necroptosis (including necrotic and apoptotic cells) into tumors in mice activated killer T cells, leading to the targeting of malignant tumors and a reduction in their growth rate [68]. Components of the LTBR-related signaling complex, including TRAF2, TRAF3, NIK, IKK1, and IKK2, are involved in the coupling of LTBR to NF-κB [69]. AdipoR1 acts as an inhibitor of LTBR activation of the NF-κB pathway [70]. Inhibition of the LTBR signaling pathway effectively suppresses atypical NF-κB activation and reduces TGFβ signaling in airways. This inhibition also promotes regeneration by preventing epithelial cell death and activating the WNT/β-catenin signaling pathway in alveolar epithelial precursor cells [71]. Additionally, LTBR plays a crucial role in immune system development and immune response. At the cellular level, ligand-bound LTBR activates the proinflammatory NF-κB pathway [72]. These studies offer valuable insights into understanding the mechanisms of LTBR in the immune microenvironment and cancer.

Stemness refers to the ability of normal cells to differentiate into the various cell types that comprise the human body. The gradual loss of cell differentiation capacity and the acquisition of stem-like characteristics are recognized as significant factors driving tumor progression [73]. Researchers have found that metastatic tumors are often similar to stem cells. In addition, this tumor stemness index can help researchers effectively identify novel targets for anti-cancer drugs, which can help researchers develop novel therapies to inhibit tumor progression [74]. We found that LTBR was significantly correlated with the stemness index in several tumors, such as LTBR and stemness index were significantly negatively correlated in TGCT, SARC, KIPAN, DLBC, and COAD, and LTBR and stemness index were significantly positively correlated in UVM, THYM, LGG, LUSC, and STEC. In addition, we used CellMine, GDSC, and CTRP databases to identify several drugs with sensitivity to LTBR. These provide an initial direction for the development of anti-cancer drugs.

In summary, we investigated the biological functions and prognostic relevance of LTBR in diverse cancers. Our comprehensive analysis explored the functional implications of altered expression levels in prognosis, genetic alterations, tumor immunity, and expression regulation across multiple cancer types. Notably, we identified LTBR as a potential target for cancer immunotherapy and a marker of immune infiltration and poor prognosis. This study offers new possibilities for the diagnosis and treatment of cancer patients, instilling hope for improved outcomes.

## MATERIALS AND METHODS

### Expression analysis of LTBR

With the development of bioinformatics, more and more researchers are utilizing data from public databases to explore the potential pathogenesis of diseases. We downloaded the uniformly normalized pan-cancer dataset from the UCSC-XENA (https://xenabrowser.net/) database. In addition, we downloaded the copy number variation (CNV) dataset from GDC (https://portal.gdc.cancer.gov/) at the gene level of level4 for all Cancer Genome Atlas Program (TCGA) samples processed by GISTIC software, and we integrated the copy number data and gene expression data of the models. Many studies have explored cancer-related biomarkers using the TCGA database [75–77]. Next, the expression levels of LTBR in tumor cell lines were obtained from the Cancer Cell Line Encyclopedia (CCLE; https://sites.broadinstitute.org/ccle/) database. The expression levels of LTBR in individual normal tissues were obtained from the Genotype-Tissue Expression (GTEx; https://commonfund.nih.gov/GTEx) database. Alternative polyadenylation (APA) is a mechanism that regulates eukaryotic gene expression and produces isomers of different 3′UTR lengths. Widespread APA affects posttranscriptional gene regulation of mRNA translation, stability, and localization and exhibits strong tissue specificity. We used the APAatlas database (https://hanlaboratory.com/apa/), which systematically identified APA events in 9475 samples from 53 human tissues [78], to study their association with multiple traits and LTBR expression across tissues. The Human Protein Atlas (HPA) database (Human Protein Atlas https://proteinatlas.org) was used to check the protein expression level [79]. Immunofluorescence staining images were also used to show the subcellular localization of LTBR in cancer cells.

### Prognostic analysis of LTBR

Log-rank test analysis and univariate Cox regression (UniCox) analysis were used to explore the effect of LTBR on patient survival in pan-cancer. Many studies frequently use them to establish the predictive profile of tumor-associated biomarkers [80–82]. Log-rank test analysis was used to assess the effect of LTBR on overall survival (OS). UniCox analysis was used to determine OS, disease-specific survival (DSS), the disease-free interval (DFI), and the progression-free interval (PFI).

### Analysis of LTBR in clinical stages, immune subtypes, and molecular subtypes

The expression values of LTBR in each clinical stage were extracted for analysis. The TISIDB (http://cis.hku.hk/TISIDB/) database [83] was used to obtain the immune subtypes of LTBR in 33 cancers, including C1 (wound healing). The database was also used to analyze the molecular subtypes of LTBR in 13 tumors, including C1 (wound healing), C2 (IFN-gamma dominant), C3 (inflammatory), and C4 (lymphocyte depleted), C5 (immunologically quiet), and C6 (TGF-b dominant). Molecular subtype profiles of LTBR in 13 tumors were also analyzed using this database.

### Correlation analysis of LTBR with immunomodulatory genes, immune checkpoint genes, and RNA-modified genes

LTBR, 150 immunoregulatory (chemokine (41), receptor (18), MHC (21), Immunoinhibitor (24), Immunostimulator (46)) marker genes, 60 immune checkpoint-related genes (Inhibitory (24), Stimulatory (36)) were extracted. Stimulatory (36) marker genes and 44 RNA modified (m1A (10), m5C (13), m6A (21)) genes expression data were in each sample. The correlation of LTBR with immunoregulatory, immune checkpoint, and RNA-modified genes was calculated separately using the Spearman algorithm.

### Immune infiltration analysis

Many studies have performed immune cell infiltration analysis to understand the correlation between genes and tumor immune cells [84–86]. The correlation analysis data of LTBR with immune cells in terms of each algorithm was obtained from the TIMER2.0 database (http://timer.cistrome.org/). The results were finally visualized using the “ggplot2” R package. The ESTIMATE R package was used to calculate the ESTIMATEScore, ImmuneScore, and StromalScore for each tumor and to calculate the correlation coefficients between the LTBR and these three scores.

### Correlation analysis of LTBR and cancer-associated functional States at the single-cell level

The CancerSEA database (http://biocc.hrbmu.edu.cn/CancerSEA/) collects 72 single-cell datasets. It provides 14 cancer-related functional states (angiogenesis, apoptosis, cell cycle, cell differentiation, DNA damage, DNA repair, EMT, cellular hypoxia, inflammation onset, cancer cell invasion, metastasis, proliferation, cell resting, and stem cell properties). The database was used to analyze the correlation of LTBR with 14 cancer-related functional states in different cancers and visualized using the “ggplot2” R package.

### Correlation analysis of LTBR and tumor stemness index

We obtained DNAss tumor stemness scores for each tumor calculated based on methylation profiles from a previous study [74]. We integrated the samples’ stemness index and LTBR gene expression data and calculated the correlation between them using the Spearman algorithm. We also used EREG-METHss, DMPss, and ENHss tumor stemness scores to validate the results further.

### Analysis of mutations, TMB and MSI

We used the CBioPortal database (https://www.cbioportal.org) to analyze the mutation characteristics and mutation location of LTBR in RSEM-normalized mRNA expression data. Illumina methylation 450 k level 3 data were downloaded from the TCGA database. The relationship between LTBR expression levels and methylation levels in the promoter region of each cancer was analyzed and visualized using the R package “ggplot2”. Correlations between LTBR gene expression and TMB or MSI in different tumors in TCGA were examined by Spearman’s test and visualized with the “fmsb” R software package.

### Analysis of MMR Genes, DNA methyltransferase

DNA mismatch repair (MMR) is an intracellular mismatch repair mechanism. Loss of function of key genes in this mechanism leads to unrepaired DNA replication errors and, consequently, higher somatic mutation production [87, 88]. DNA methylation, as a form of chemical modification of DNA, can cause changes in chromatin structure, DNA conformation, DNA stability, and the way DNA interacts with proteins, thereby controlling gene expression. It is covalently bonded to a methyl group at the cytosine 5’ carbon position of genomic CpG dinucleotides by the action of DNA methylation transferase [89–91]. Here we analyzed the correlation of gene expression with four methyltransferases (DNMT1, DNMT2, DNMT3A, and DNMT3B).

### GO, KEGG and GSEA

The top 100 genes with co-expression of LTBR in 33 tumors were obtained from the GEPIA2 database (http://gepia2.cancer-pku.cn/) and analyzed by GO and KEGG using the “clusterprofiler” R package analysis where GO includes molecular function (MF), cellular component (CC), and biological process (BP). In addition, we downloaded the hallmark gene set from Molecular Signatures Database (MSigDB), which contains 50 important pathways affecting cancer. The Normalized Enrichment Score (NES) and False Discovery Rate (FDR) of LTBR were calculated for each path in each tumor. GSEA was performed using the R packages “clusterProfiler” and “GSVA,” and the results are summarized in bubble plots drawn by the R package “ggplot2”.

### Drug sensitivity analysis

To develop relevant drugs against target genes, we explored the relationship between LTBR expression and drug sensitivity using the CellMine database (https://discover.nci.nih.gov/cellminer). In addition, to expand the study, we also obtained mRNA expression and drug sensitivity data from GDSC (https://www.cancerRxgene.org) and CTRP (https://portals.broadinstitute.org/ctrp.v2.1/). Correlation analysis was performed to obtain the correlation between gene mRNA expression and drug IC50.

### Cell culture

The following cancerous cell lines: C3A, Caco2, HepG2, SW480, NH4, SCC25, as well as normal cell lines (293T, HOK) were preserved in DMEM media, which was enhanced with 10% (v/v) fetal calf serum and 1% (v/v) penicillin G/streptomycin (15240062 Gibco, USA). The conditions were kept stable at 37°C under an atmosphere of 5% CO2.

### Quantitative real-time PCR

Total RNA was isolated from cells utilizing TRIzol reagent, followed by reverse transcription into cDNA with the use of the PrimeScript RT Reagent Kit (TaKaRa, Japan). The quantitative PCR was executed using FastStart Universal SYBR Green Master Mix (ROX) (Roche, Switzerland) on the Roche LightCycler 480 II Real-Time PCR system (Roche, Switzerland). Gene expression levels were measured in triplicate. The primers used for qPCR experiments were as follows: Human LTBR, forward, 5′-GAAGGGTAACAACCACTGC-3′; reverse, 5′-CTTGGTTCTCACACCTGGT-3′. Human GAPDH, forward, 5′-TCAAGATCATCAGCAATGCC-3′; reverse, 5′-CGATACCAAAGTTGTCATGGA-3′. Relative gene expression levels were calculated using the 2^ΔΔCt^ method. All experiments were conducted in triplicate.

### Western blot

The cells were washed using an ice-cold phosphate-buffered saline (PBS) and then lysed with the Membrane and Cytosol Protein Extraction Kit (20127ES60 Yeasen, China). The kit was further supplemented with protease (20124ES03 Yeasen, China) and phosphatase inhibitors (20109ES05 Yeasen, China). The concentration of the proteins was determined via the BCA Protein Assay Kit (20201ES76 Yeasen, China), and the process was conducted in accordance with the manufacturer’s instructions. The proteins were segregated on 4–20% Bis-Tris gels (Genscript, China), subsequently being transferred onto polyvinylidene difluoride (PVDF) membranes (Millipore ISEQ00010, China). The membranes were then blocked using 5% non-fat milk in Tris-buffered saline with Tween 20 (TBST) at room temperature for 1 hour. Primary antibodies were diluted in 0.5% non-fat milk in TBST and were then allowed to incubate with the membrane at 4°C overnight: Anti-LTBR antibody (Absin abs146148, 1:1,000), Anti-GAPDH (Absin abs830030, 1:2,000). After three washes with TBST, the membrane was incubated with horseradish peroxidase (HRP)-conjugated secondary antibodies (34201ES60 Yeasen, China) in TBST at room temperature for 1 hour. The immunoreactive bands were then visualized utilizing the Enhanced Chemiluminescence (ECL) Western Blotting Substrate (36208ES60 Yeasen, China).
